# Oral manifestations of vitamin B12 deficiency associated with pernicious anemia: A case report

**DOI:** 10.1016/j.ijscr.2024.109931

**Published:** 2024-06-22

**Authors:** S. Boukssim, S. Chbicheb

**Affiliations:** Department of Oral Surgery, Faculty of Dentistry, Mohammed V University, Morocco

**Keywords:** Pernicious anemia, Oral manifestations, Vitamin B12, Case report

## Abstract

**Introduction and importance:**

Vitamin B12 deficiency can manifest through various oral manifestations such as glossitis, glossodynia, recurrent ulcers, cheilitis, dysgeusia, lingual paresthesia, burning sensations, and pruritus. These oral signs can serve as early indicators of systemic conditions such pernicious anemia.

**Case presentation:**

A 67 year old northern African female presented at the oral surgery service with complaints of a sore mouth and difficulty eating certain types of food. Her medical history revealed hypothyroidism and no history of gastrectomy. She was diagnosed with pernicious anemia in 2014 and is under hydroxocobalamin injection 5000μg/month since then. Dental history indicated extraction of all teeth, and in 2014, the patient was diagnosed with oral lichen planus. There were no contributory oral habits. Intraoral examination revealed a band like erythematous lesion on the palate with two superficial ulcerations, diagnosed as related to her pernicious anemia. The patient was prescribed a mouthwash containing sodium bicarbonate and corticosteroid to reduce inflammation and alleviate pain. A low level laser therapy was also considered to reduce the burning sensations.

**Clinical discussion:**

Pernicious anemia (PA) is an autoimmune disease characterized by the gradual atrophy of the gastric mucosa, predominantly affecting the body and fundus of the stomach, leading to vitamin B12 deficiency. Its insidious onset often masks its presence. Patients have no anemic symptoms. However, they can present with oral manifestations related to vitamin B12 deficiency. Those oral signs can precede hematological symptoms helping in early diagnosis of PA.

**Conclusion:**

Dentists and other oral health care providers must be aware of this condition and its oral manifestations. Investigating vitamin B12 levels should be considered in patients presenting with oral ulcers, oral erythema or burning sensations without an apparent origin.

## Highlights

• Pernicious anemia is a rare autoimmune disease responsible for a deficiency in vitamin B12.
• Vitamin B12 plays a crucial role in nutrition, and its deficiency is associated with various oral manifestations.
• Dentist play a vital role in the early diagnosis of vitamin B12 deficiency and its underlying causes

## 1 Introduction

Vitamin B12 plays a crucial role in oral health, and its deficiency can lead to a spectrum of oral manifestations. These include glossitis, glossodynia, recurrent ulcers, cheilitis, dysgeusia, lingual paresthesia, burning sensations, and pruritus [1,2].

Pernicious anemia (PA) serves as a primary cause accounting for 20 %–50 % of documented cases of vitamin b12 deficiency in adults [3].

These oral changes may precede systemic symptoms of pernicious anemia, underscoring their importance as early indicators of pernicious anemia [1].

Here, we present a case report of patient with already diagnosed pernicious anemia who presented with oral manifestations.

In this case we describe the oral manifestations related to pernicious anemia, review the literature regarding its symptoms, diagnostic methods and treatment and underscore the importance of oral health in diagnosing systemic conditions.

This case report adheres to the SCARE Criteria [4].

## 2 Case report

A 67 year old northern African female presented at the oral surgery service with a chief complain of sore mouth, burning sensations and difficulty in eating certain types of food.

The medical history of the patient revealed the presence of hypothyroidism treated by levothyroxine sodium and no history of gastrectomy.

It also revealed she was diagnosed with pernicious anemia and is under hydroxocobalamin injection 5000μg/month since 2014.

The diagnosis of PA was initially considered based on past oral manifestations, including oral pain and white lesions on the internal side of the cheek, which were diagnosed as lichen planus.

After being diagnosed with vitamin B12 deficiency and receiving monthly injections to correct it, the patient experienced an improvement in oral lesions. However, despite ongoing vitamin B12 treatment, the lesions recurred, though with less intensity.

The current level of vitamin B12: 2000 pg/ml; this high level is explained by the monthly hydroxocobalamin injections.

The personal history revealed no oral habits that would contribute to the complaint.

**Fig. 1:**
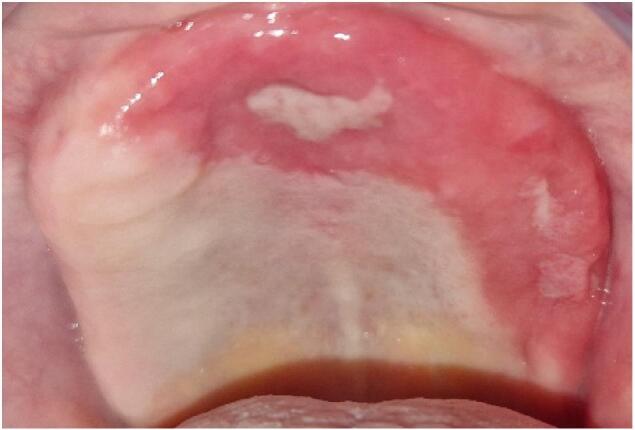
Erythematous lesion involving the palate with two ulcerations.

The intraoral examination revealed a band like erythematous palate lesion with two superficial ulcerations (Fig. 1), diagnosed as linked to her pernicious anemia.

A biopsy of the ulceration revealed a gingival mucosa lined by mildly hyperplastic squamous epithelium showing massive exocytosis of mononuclear inflammatory cells. The chorion contains a dense lymphoplasmacytic infiltrate forming diffuse sheets without a clear border in depth.

For oral management of the lesion, a mouthwash containing sodium bicarbonate and corticosteroid was prescribed to reduce inflammation and alleviate pain. After two weeks of use, a slight reduction in redness and ulcerations was observed. However, the patient continued to experience a burning sensation in the mouth. Consequently, low-level laser therapy (LLLT) sessions were indicated. LLLT utilized a spectral range of 630 nm, with high irradiance reaching up to 100mw/cm2 once a week.

**Fig. 2:**
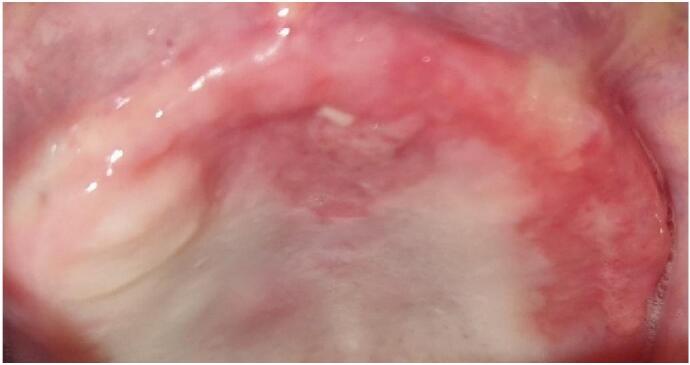
Improvement of the lesion after 6 LLLT sessions.

After 6 sessions, significant improvement was observed (Fig. 2), and the patient reported a reduction in burning sensation from 9/10 to 3/10 on the Visual Analog Scale (VAS).

To assess the impact on oral health-related quality of life, the Oral Health Impact Profile-14 (OHIP-14) was administered before and after laser therapy. The total OHIP-14 score decreased from 54 to 28, indicating a notable improvement in functional limitation, pain, discomfort, and overall quality of life.

Although the patient initially benefited from LLLT, there has been a lapse in adherence to the therapy schedule following the reduction in burning sensation.

## 3 Discussion

Vitamin B12 is an important nutritional components that affect oral health. The main cause of this deficiency is pernicious anemia.

Pernicious anemia (PA) is an autoimmune disease characterized by the gradual atrophy of the gastric mucosa, predominantly affecting the body and fundus of the stomach. This condition leads to a decrease in the number of parietal cells (PA) responsible for producing the intrinsic facto (IF), necessary for the absorption of cobalamin, commonly known as vitamin B12. [5]. Two types of autoantibodies, intrinsic factor antibodies (IF) and parietal cell antibodies (PC), target the binding site of cobalamin as well as interfere with binding of IF to the epithelium of ileal mucosa decreasing the absorption of cobalamin [6].

The insidious onset progression of PA often masks its presence. Patients have no anemic symptoms since they become acclimatized to the subtle nature of the disease. Generally, it takes 10–12 years to clinically develop symptomatic PA [7]. The underlying disease might go undetected unless full blood count is investigated. [6]

However, patients with PA may present with nonspecific symptoms associated to anemia such as lethargy, inability to concentrate, headache, and cardiac manifestations such as palpitations and chest pain, particularly observed in elderly patients [6].

The diagnosis of PA relies on demonstration of megaloblastic anemia, low serum vitamin B12 levels, gastric atrophia, along with the presence of antibodies to gastric PC or IF. [5]

Despite these diagnostic criteria, PA diagnosis can be challenging due to various morphological mimics, and its diverse clinical presentations [6].

In fact serum cobalamin may be falsely normal or even elevated in 22 %–35 % of patients with PA [8].

In such instances, additional tests measuring fasting homocysteine and serum methylmalonic acid (metabolites of the cobalamin) become imperative. Moreover, the assessment of PC and IF antibodies might not yield positive results in about 30 % of PA cases [6].

In such cases, elevated fasting serum gastrin levels can hint toward PA. In challenging cases with a strong suspicion of deficiency, a trial of vitamin B12 injections may be considered. While reticulocyte count typically improves within days, full recovery may take up to two months. [6]

Vitamin B12 is an important nutritional components that affect oral health. It's deficiency can manifest through oral mucosal changes which have been reported in 50–60 % of patients with megaloblastic anemia [9].

These changes may precede hematological symptoms and indicate systemic health problems [10].

Oral manifestations include burning sensations of the tongue, lips, and buccal mucosa alongside focal or diffuse erythema and mucosal atrophy [11].

A more specific oral manifestation is the so called “Hunter glossitis” characterized by a shiny, lacquered tongue that assumes a “beefy” red color [2].

In this case, the patient presented with diffuse erythema of the palate associated with two superficial ulcerations. The diagnosis of pernicious anemia of the patient was already established, however in some cases, the oral manifestations can lead to the diagnosis of the disease.

Hence, dentists play a crucial role in identifying theses oral changes. They should consider pernicious anemia, as a possible diagnosis, in patients presenting erythematous macules of the oral mucosa, irrespective of accompanying lingual signs and symptoms [9].

A comprehensive history should include questions regarding dyspnea, weakness, paresthesia, ataxia and gastrointestinal symptoms. A complete blood count, which includes red blood cell indices, should be obtained in all cases. [9]

The treatment of B12 deficiency primarily involves intramuscular injections of cyanocobalamin, hydroxocobalamin or methyl cobalamin, with regular monitoring Vitamin B12 levels and iron deficiency [12].

Hydroxocobalamin is a natural form of Vitamin B12 and is said to have better tissue uptake and storage than the other forms [12].

The most frequently applied intramuscular injection dose is 1000μg, administered daily to weekly initially, followed by monthly injections for maintenance of adequate B12 status [13].

In this case, the patient benefited from hydroxocobalamin injection 5000μg/month.

Intramuscular injections have traditionally been the primary method for treating vitamin B12 deficiencies. However, new administration alternatives like oral, sublingual, and nasal options have arisen [14]. These alternatives are particularly beneficial for elderly patients, as they offer a way to avoid the discomfort, inconvenience, and expense of monthly injections [12,14].

A narrative review on oral and nasal vitamin B12 therapy in the elderly [14], along with insights from the ‘CARE B12’ group [15], strongly advocates for incorporating oral vitamin B12 replacement into clinical practice. Despite the limitations of the studies, they recommend a daily dose of 1000 μg of oral cyanocobalamin for lifelong supplementation [14,15].

The development of various commercial forms of intranasal cyanocobalamin, including spray-dried powders and nasal gels, has renewed interest in this administration route. A weekly dose of 500 μg/0.1 ml intranasally has been approved as a therapy for vitamin B12 deficiency, including pernicious anemia [14].

Oral supplementation becomes the preferred choice when intramuscular injections are impractical due to conditions like coagulopathy or the use of anticoagulants [12,14]. Elderly patients, particularly those with sarcopenia, often prefer oral therapy over injections due to the discomfort and challenges associated with intramuscular injection administration [12,14].

For oral management of the lesions, although the efficacy of topical corticosteroids is not strongly supported by evidence, they remain the most frequently prescribed drugs in oral medicine and are widely utilized to treat various immune-driven inflammatory mucosal lesions [16]. In this case, the application of topical corticosteroid as a mouthwash yielded minimal results.

Low level laser therapy has emerged as an important innovation in pain management and finds widespread application in clinical settings, particularly for conditions such as oral mucositis, oral lichen planus and neuropathic orofacial pain [17]. It is a recommended complementary treatment option when pharmacotherapy alone is not sufficient [18].

The optical spectral range used in LLLT is between 600 and 1100 nm and higher dose irradiance (up to 50 mW/cm2) is beneficial for nerve inhibition and pain relief [19]. The intervention frequency is typically recommended as 1 or 2 times per week.

In this case, the optical spectral range of LLLT was administered at an optical spectral range of 630 nm with an irradiance of 100mw/cm2 once per week.

Regular monitoring is essential in PA management to ensure treatment adherence, monitor for complications such as gastric tumors via biannual upper endoscopy surveillance, manage coexisting autoimmune conditions, and provide nutritional guidance. Collaborative care involving primary care physicians, specialists, and dietitians is crucial for optimizing outcomes in patients with PA.

## 4 Conclusion

PA presents a diagnostic challenge due to its diverse clinical manifestations, often leading to delays in its identification and management.

Therefore, in the presence of oral sign and symptoms, such oral ulcer, erythematous mucositis and pale oral mucosa without obvious apparent origin, evaluating the levels of serum B12 should be consider by dentists.

## Patient consent

Written informed consent was obtained from the patient for publication of this case report and accompanying images. A copy of the written consent is available for review by the Editor-in-Chief of this journal on request.

## Ethical approval

Ethical approval is exempt at our institution. Not required for case reports.

Comité d'Ethique pour la recherche Biomédicale (CERB)- Mohamed V University.

## Funding

None.

## Author contribution

Sara Boukssim is first author: Diagnostic workup, Drafting the manuscript and Literature research.

Saliha Chbicheb: Supervision and critical revision.

## Guarantor

Sara Boukssim.

## Research registration number

Elsevier does not support or endorse any registry.

## Declaration of competing interest

No conflict of interest.

## Appendix A Supplementary data

Supplementary material Image 1
