# Supplementary material for: Oral manifestations of vitamin B12 deficiency associated with pernicious anemia: A case report
Source: Int J Surg Case Rep. 2024 Jun 22;121:109931. doi: 10.1016/j.ijscr.2024.109931 (PMC11254220; doi:10.1016/j.ijscr.2024.109931)
Supplement: Supplementary file 1 — Supplementary material [file mmc1.docx]

**The pain scale assessment**

**The visual scale assessment**

0 |-----------------------------------------------------------| 10

No Pain Worst Pain

**The oral health impact profile questionnaire (short version)**

**The OHIP-14:** it includes the following questions, with responses typically rated on a scale from 0 (never) to 4 (very often):

1. **Functional Limitation**:
   - Have you had trouble pronouncing any words because of problems with your teeth, mouth, or dentures?
   - Have you felt that your sense of taste has worsened because of problems with your teeth, mouth, or dentures?
2. **Physical Pain**:
   - Have you had painful aching in your mouth?
   - Have you found it uncomfortable to eat any foods because of problems with your teeth, mouth, or dentures?
3. **Psychological Discomfort**:
   - Have you been self-conscious because of your teeth, mouth, or dentures?
   - Have you felt tense because of problems with your teeth, mouth, or dentures?
4. **Physical Disability**:
   - Has your diet been unsatisfactory because of problems with your teeth, mouth, or dentures?
   - Have you had to interrupt meals because of problems with your teeth, mouth, or dentures?
5. **Psychological Disability**:
   - Have you found it difficult to relax because of problems with your teeth, mouth, or dentures?
   - Have you been a bit embarrassed because of problems with your teeth, mouth, or dentures?
6. **Social Disability**:
   - Have you been a bit irritable with other people because of problems with your teeth, mouth, or dentures?
   - Have you had difficulty doing your usual jobs because of problems with your teeth, mouth, or dentures?
7. **Handicap**:
   - Have you felt that life in general was less satisfying because of problems with your teeth, mouth, or dentures?
   - Have you been totally unable to function because of problems with your teeth, mouth, or dentures?

Each question is scored from 0 to 4:

- 0 = Never
- 1 = Hardly ever
- 2 = Occasionally
- 3 = Fairly often
- 4 = Very often
